# Clinical response to dabrafenib plus trametinib in BRAF V600E mutated papillary craniopharyngiomas: a case report and literature review

**DOI:** 10.3389/fonc.2024.1464362

**Published:** 2024-11-27

**Authors:** Paul Hanona, Daniel Ezekwudo, Joseph Anderson

**Affiliations:** Beaumont Hospital, Beaumont Health, Royal Oak, MI, United States

**Keywords:** papillary, craniopharyngioma, BRAF mutation V600E, dabrafenib, trametinib

## Abstract

Papillary craniopharyngiomas are rare tumors prevalent to the precision oncology world due to their high rate of BRAF V600E mutations. Symptoms include vision loss, neuroendocrine dysfunction, and cognitive dysfunction. Treatment involves an interdisciplinary approach with surgery, radiation, and systemic treatment. Recent attention has been directed toward targeted therapy in this space, especially with targets to the BRAF V600E mutated pathway. Focusing on this pathway could solidify future standards of care treatment. A 61-year-old male came in with bilateral homonymous hemianopsia. This prompted a brain MRI that showed a bilobed centrally cystic peripherally enhancing sellar and suprasellar mass with mass effect on the left greater than right optic chiasm and nerves. He underwent a primary resection of the suprasellar cystic tumor, and it was revealed that he had papillary craniopharyngioma. Three months later, he represented with visual defects, and repeat MRI showed cystic recurrence with compression of the optic chiasm. He underwent an endonasal resection of the middle fossa tumor; pathology, this time, showed a BRAF V600E mutated papillary craniopharyngioma. Nine months later, another recurrence happened, and the patient was started on BRAF and MEK inhibitors: dabrafenib (75 mg BID) and trametinib (2 mg daily). The patient has had clinical improvement of visual symptoms and is currently continuing this treatment. He was last seen in October of 2024, and he is clinically stable. The use of targeted therapies is an evolving space for BRAF V600E mutated papillary craniopharyngiomas. This is a case showing improvement of a craniopharyngioma after treatment with BRAF and MEK inhibitor combinations. The role of BRAF and MEK inhibitor combinations continues to evolve in this space.

## Introduction

Craniopharyngiomas are slow-growing brain tumors that originate from Rathke’s pouch remnants. They are incredibly rare with only 350 cases a year in the United States (1). They have a bimodal age distribution and occur equally in both men and women (2). They can arise near the pituitary stalk, within the sella, the third ventricle, and optic chiasm (3). Tumor topography, especially in regards to its relationship with the hypothalamus, can indicate tumor recurrence rates (4). They break down into two types, either adamantinomatous or papillary. Adamantinomatous types have mutations in CTNNB1, while papillary types have mutations in BRAF. Both types are overwhelmingly mutated with 96% of adamantinomatous types having CTNNB1 mutations and 94% of papillary types having BRAF V600E mutations. They have similar overall survival. The 10-year overall survival ranges between 80% and 96% (5).

Symptoms are challenging to identify due to the slow-growing nature of craniopharyngiomas. The most common symptoms include headaches, visual field deficits, endocrine alternations, and mental distrubances (6). Diagnosis involves MRI of the brain and or CT of the brain. A mass is usually seen that compresses nearby structures.

Calcifications are often seen (7). Endocrine testing for abnormalities in pituitary hormones is also crucial (6).

Treatment has traditionally involved surgery and radiation, with a more recent addition of targeted therapy. Neuroimaging alone can suggest BRAF mutant papillary types with a representative feature like lack of calcification. In these cases, biopsy, instead of aggressive surgery, is preferred, and the patient can be put on first-line targeted therapy with BRAF/MEK inhibitors. If neuroimaging suggests an adamantinomatous types, aggressive resection is preferred due to mass effect causing symptoms commonly being the presenting sign (8). Monitoring of endocrine function, edema, and hydrocephalus is also crucial during this period.

Targeted therapy is a fundamental tenet in the treatment of papillary craniopharyngiomas. All papillary craniopharyngiomas should be tested for BRAF V600E. Surgical resection of the tumor is the gold standard. First-line treatment of newly diagnosed craniopharyngiomas after resection is BRAF/MEK inhibition. This involves four to six cycles of targeted therapy and then reassessment for the need for RT, surgery, or continued therapy with BRAF/MEK inhibition. These agents usually include dabrafenib plus trametinib or vemurafenib plus cobimetinib (9). This is an ongoing area of research. This case report seeks to add to the literature that shows clinical improvement of craniopharyngiomas with BRAF/MEK inhibition.

## Case description

This is a case of a 61-year-old male who first presented with changes in his vision in May 2022. A summary timeline of the patient's case can be found in **Table 1**. He has a history of hypertension, sarcoidosis, prediabetes, and sinus bradycardia. He had no surgeries up until this point. He is a never smoker and a never drinker. He had no relevant family history. Physical exam signs were largely remarkable for bilateral homonymous hemianopsia. Examination revealed visual acuity of 20/25 + 2 in the right eye and 20/200 + 1 with pinhole to 20/80−2 in the left eye. Brain MRI showed a bilobed centrally cystic peripherally enhancing sellar and suprasellar mass with mass effect on the left greater than right optic chiasm and nerves. He underwent an endonasal resection of the middle fossa tumor. Pathological results were indeterminant. His vision improved following his surgery.

**Table 1:** Timeline of patient’s clinical history.

Date	Clinical	Radiological	Treatment
May 2022	Bilateral hemianopsia	Brain MRI showing a suprasellar mass	Endoscopic resection of craniopharyngioma with pathology being indeterminant
September 2022	Bilateral hemianopsia	Brain MRI showing a mass resembling a craniopharyngioma	Endoscopic resection of craniopharyngioma with pathology showing papillary craniopharyngioma, CNS WHO grade 1 with a BRAF V600E mutation
June 2023	Bilateral hemianopsia	Brain MRI showing recurrence of the craniopharyngioma	Patient is started on dabrafenib and trametinib
August 2023	Bilateral hemianopsia worsening	Brain MRI showing slight increase in the size of the craniopharyngioma	Third revision endonasal resection with residual mass remaining
September 2023	Stable bilateral hemianopsia	Not available	Resumed dabrafenib and trametinib
October 2024	Stable bilateral hemianopsia	Not available	Continuing dabrafenib and trametinib

**Figure 1:**
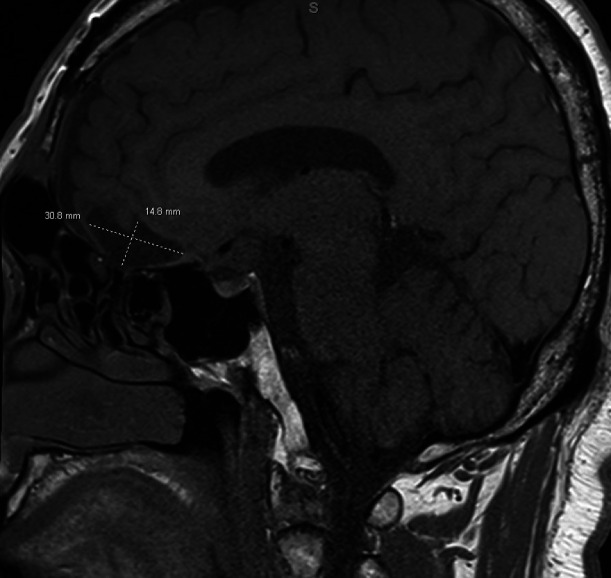
Sagittal brain MRI showing a 10.3 mm × 14.6 mm × 17 mm cystic mass in the suprasellar area with enhancing mural nodules, most likely related to craniopharyngioma, causing mass effect on the optic chiasma.

**Figure 2:**
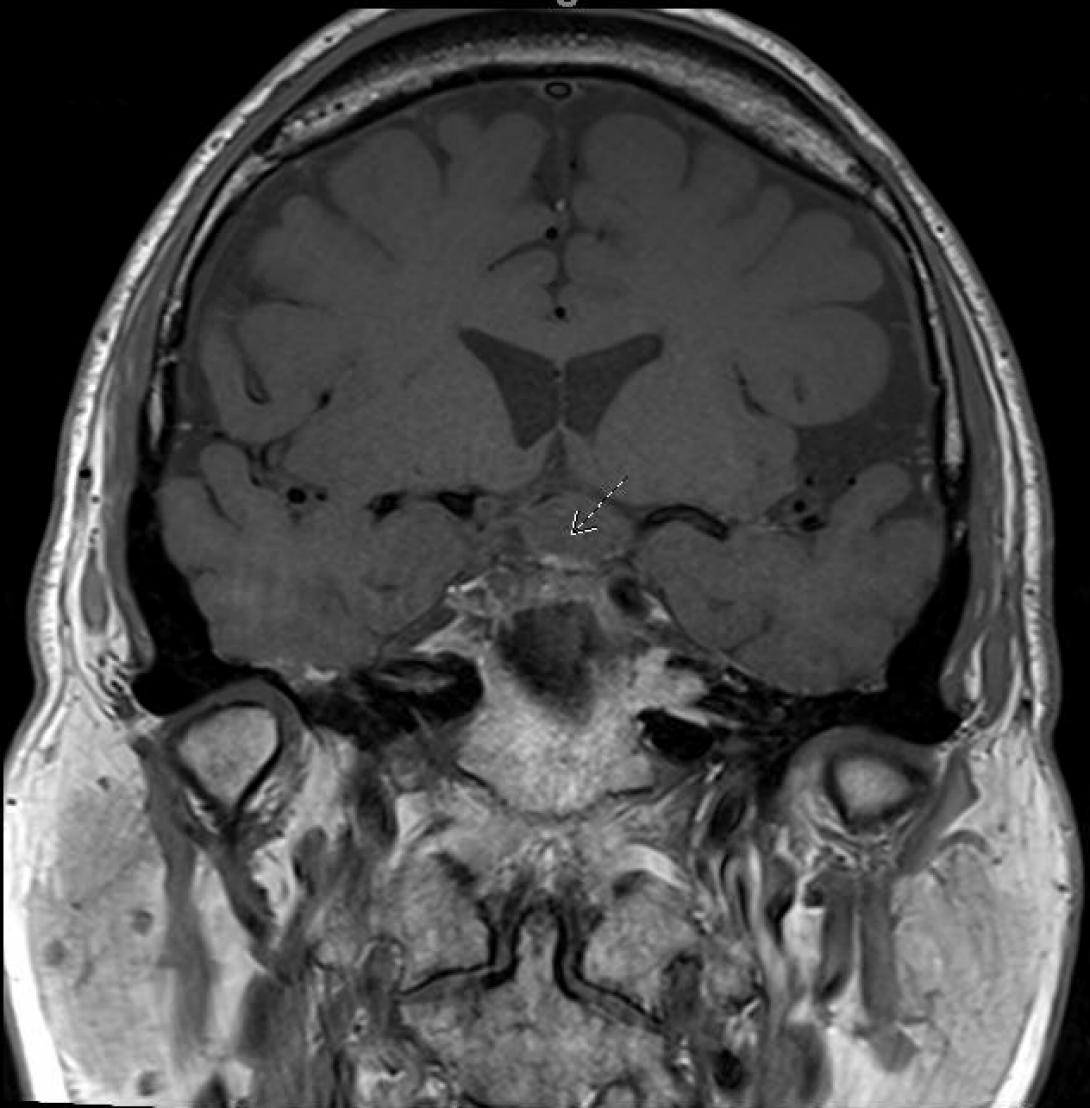
MRI coronal-transinfundibular showing a cystic mass (white arrow) in the suprasellar area with enhancing mural nodules, most likely related to craniopharyngioma, causing mass effect on the optic chiasma.

In August 2022, the patient presented again with visual deficits. Repeat MRI showed a 10.3 mm × 14.6 mm × 17 mm cystic mass in the suprasellar area with enhancing mural nodules, most likely related to craniopharyngioma that is causing mass effect on the optic chiasma (**Figures 1**, **2**). He underwent an endoscopic endonasal transplanum transtuberculum approach to the middle fossa skull base with a resection of the middle cranial fossa skull base tumor. Pathology revealed papillary craniopharyngioma, CNS WHO grade 1. BRAF V600E mutation was identified. Subsequently, a CSF leak was present, and he underwent a CSF leak repair. He followed up with the surgical team who monitored for symptoms of clinical relapse. Importantly, the patient never went for radiation.

**Figure 3:**
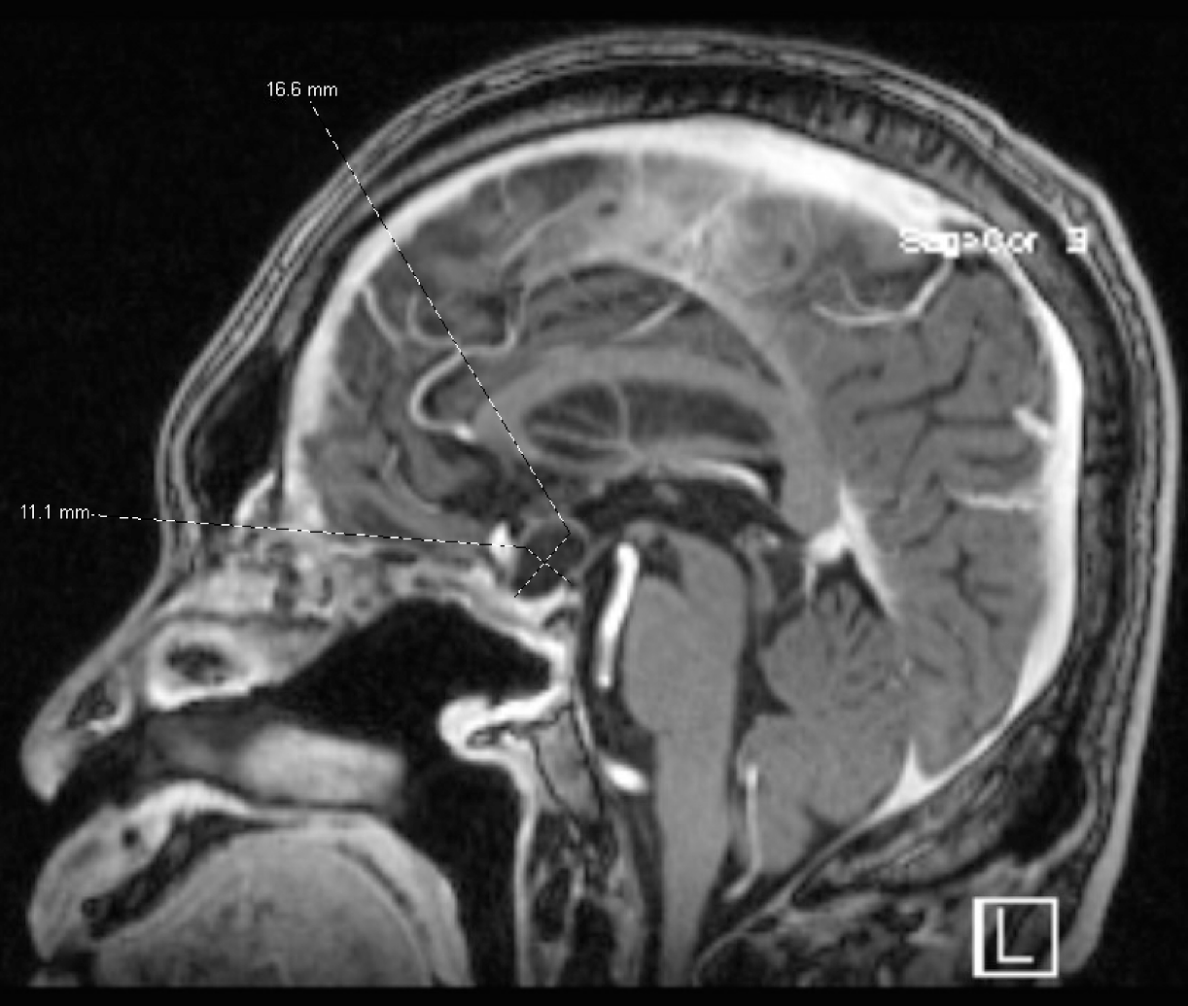
Brain MRI in August 2023 showing a suprasellar cystic brain mass consistent with a craniopharyngioma.

Eight months after this second surgery, he had his first visit with an oncologist in June 2023. He again was having visual deficits, and he had an MRI that showed recurrence of the craniopharyngioma. At the time, it felt too risky to go back for a third neurosurgical resection; thus, a joint decision was made to have the patient undergo trial on targeted therapy with dabrafenib and trametinib. He started on dabrafenib 150 mg twice daily and trametinib 2 mg once daily. The plan was to keep him on this targeted therapy until progression. One month later, his vision improved, but his symptoms had not completely resolved. He did develop myalgia and fatigue while on targeted therapy, but otherwise, he was tolerating the therapy well. A follow up visit in August 2023 showed that his visual symptoms had gotten worse in the right eye. A brain MRI at that time was repeated and showed growth in the suprasellar region with a cystic mass (**Figure 3**). Thus, he underwent a third neurosurgical revision with an endonasal resection of the tumor. His vision again improved almost back to normal after this third surgery. After recovery from his surgery, the patient resumed targeted therapy with dabrafenib and trametinib. Since his third surgery, he has been back on dabrafenib and trametinib. He complains of fatigue but, otherwise, is tolerating the combination well. He continues to take these medications and follows with an oncologist regularly. Recently, as of June 2024, he was working with a physical therapist for an unrelated lumbar radiculopathy. Otherwise, the patient is faring well.

## Discussion

Craniopharyngiomas are tumors that arise along the craniopharyngeal duct. Two key mutations in the CTNNB1 and BRAF V600E mutations lead to two different histological types being the adamantinomatous and papillary types, respectively. Symptoms include visual impairment, endocrine deficiencies, and other neurological abnormalities. MRI is proven to be one of the gold diagnostic standards for craniopharyngiomas. Treatment involves neurosurgery, radiotherapy, and now an evolving role for targeted treatment. Long-term survival is common; however, quality of life continues to be a challenge with common side effects of treatment including fatigue and psychosocial deficits (10). Patients commonly complain about reduction in social and emotional functioning citing that their psychosocial status is worse than their physical health. This includes patients going through anxiety, depression, and withdrawal. Patients also complained of reduced mobility (11).

The first step of the BRAF/MEK pathway involves a growth factor binding to a cell receptor. This activates the RAS protein, which activates the BRAF protein. This active BRAF protein phosphorylates and activates the MEK protein, and activated MEK protein phosphorylates and activates the ERK protein. ERK then moves into the nucleus and activates genes that help cells proliferate. When the BRAF gene is mutated, a mutated BRAF protein results. This mutated BRAF protein is constitutively activated leading to uncontrolled cell growth per the mechanism just described (12). Drugs, like dabrafenib and vemurafenib, target this mutated BRAF protein. To avoid resistance, a drug targeting the downstream MEK protein like trametinib is also given. Currently, dabrafenib is approved for mutated BRAF V600E melanomas, non-small cell lung cancers, solid tumors that are unresectable or metastatic, and thyroid cancers (13). Trametinib has the same approvals as dabrafenib, except with the addition of ovarian carcinoma (14).

Treatments utilizing surgery and radiation both have substantial morbidity. Keeping in mind that the overwhelming majority of papillary craniopharyngioma carry a BRAF V600E mutation, efforts are made to target this pathway. New approaches with targeted therapy are being investigated. The CTNNB1 mutation pathway has no current targeted treatment. However, the BRAF V600E mutation can be either targeted with dabrafenib and trametinib or vemurafenib and cobimetinib. Our patient was treated with dabrafenib and trametinib. Several case reports suggest efficacy with dabrafenib and trametinib. One case report of a 39-year old showed that the tumor volume was reduced by 85% after only 35 days (15). Another case report also showed marked tumor reduction and even improvement in the patient’s panhypopituitarism (16). A publication shows that a 35-year-old man also had his tumor reduced in size by 95% over 21 months without any side effects (17). One patient had a complete response over 2 years (18). Patients can also be kept on this treatment for more than 2 years especially since one case report showed that the patient continued to have clinical improvement 2 years after starting the dabrafenib and trametinib (19).

Since the tumors are benign in nature, targeting them with surgery and radiation can often lead to more morbidity than necessary. A summary of various studies pertaining to treatment of BRAF mutated craniopharyngiomas can be found in **Table 2**. If there was a way to introduce targeted treatment with BRAF and MEK inhibitors early on as neoadjuvant treatment, that could potentially reduce the morbidity from ensuing surgery and radiation (20). One case report showed that a 39-year old with a BRAF V600E mutated craniopharyngioma first received neoadjuvant dabrafenib and trametinib, then received definitive radiosurgery. The authors theorize that neoadjuvant targeted treatment could take patients who are poor surgical candidates and turn them into a better surgical candidate if the original tumor size shrinks (21).

**Table 2:** Literature review with primary results of cases analyzed for this manuscript.

Studies	Demographics	Context	Dosage	Primary result
Brastianos et al.	39-Year-old male	Stage IV	Dabrafenib 150 mg BID, trametinib 2 mg BID	Combination BRAF and MEK inhibition reduced the tumor by 85% after 35 days
Roque et al.	47-Year-old female	Unresectable tumor proved refractory to radiation	Dabrafenib 150 mg BID, trametinib 2 mg BID	Combination BRAF and MEK inhibition reduced the tumor by more than 75% by 5 months; however, the patient had permanent panhypopituitarism
Nussbaum et al.	35-Year-old male	Post subtotal resection	Dabrafenib 75 mg BID, trametinib 2 mg BID	Combination BRAF and MEK inhibition reduced tumor by 95% over 21 months
Wu et al.	60-Year-old female and 60-year-old male	Post subtotal resection for both cases	Dabrafenib 150 mg BID, trametinib 2 mg BID	Combination BRAF and MEK inhibition in the 60-year-old female leads to a complete response for 2 years; in the male, the same combination showed a 20% reduction in tumor size over 1 month
Rao et al.	35-Year-old male	Post subtotal resection	Dabrafenib 150 mg BID	Single-agent BRAF inhibition led to a continued response over 2 years; however, patient had a remnant of panhypopituitarism
Khaddour et al.	39-Year-old male	Post subtotal resection	Dabrafenib 150 mg BID, trametinib 2 mg BID	Combination BRAF and MEK inhibition showed a 70% tumor reduction at 9 months; patient has been in remission for 2 years
Brastianos et al.	16 Total patients	NA	Vemurafenib 960 mg BID for 28 days, cobimetinib 60 mg daily for 21 days	Median reduction in volume of tumor was 91% over 22 months. PFS was 87% at 12 months and 58% at 24 months

NA, Not available.

Data are stronger regarding the vemurafenib and cobimetinib combination. A recent phase 2 study was done with 16 patients who had BRAF V600E mutations. BRAF/MEK inhibitor combination with vemurafenib and cobimetinib was administered in 28-day cycles. Fifteen out of those 16 patients had a durable objective partial response or better. The median reduction of tumor was 91%. Progression-free survival at 12 months was 87% and at 24 months 58%. The median number of cycles was eight cycles. Notable adverse events were rashes, hyperglycemia, and dehydration (9). Some patients have more pyrexia on dabrafenib and trametinib, and thus, switching over to vemurafenib and cobimetinib may be a better option (22). Our patient had pyrexia early with dabrafenib and trametinib, and thus, we may switch him over to vemurafenib and cobimetinib if it persists as a problem.

Regarding this patient’s case specifically, he was treated with surgery multiple times before he started on targeted treatment with dabrafenib and trametinib. The patient himself remarked on the challenges of recovering from surgery multiple times. This also had a considerable effect on his quality of life. An argument could be made that the patient could have started on targeted treatment with dabrafenib and trametinib in the neoadjuvant setting before surgery, to perhaps make surgery a one-time event as opposed to multiple surgeries being necessary. Neoadjuvant dabrafenib and trametinib as a neoadjuvant could have reduced tumor volume leading to a less morbid surgery. When the patient was finally started on the dabrafenib and trametinib, one of the limitations was the ability to only assess the patient clinically and not with more frequent imaging. For example, the patient started on targeted treatment and clinically improved for 2 months before he felt that his vision was getting worse. It could have been beneficial to see what a brain MRI would have shown after 2 months of treatment, but a brain MRI would not have been approved by his insurance. Likewise, the patient had surgery after only 2 months of being treated with targeted therapy and resumed on targeted therapy after this surgery. The question could be asked if the patient is in remission because of the targeted treatment or the surgery. Limited publications are available discussing dabrafenib and trametinib in the neoadjuvant setting. Ultimately, more research is required to address this paradigm of using targeted treatment as a neoadjuvant treatment and then deciding whether or not the patient even needs surgery or radiation.

## Data Availability

The original contributions presented in the study are included in the article/**Supplementary Material**. Further inquiries can be directed to the corresponding author.

## References

[1] Ostrom QT Cioffi G Waite K Kruchko C Barnholtz-Sloan JS. CBTRUS statistical report: primary brain and other central nervous system tumors diagnosed in the United States in 2014-2018. Neuro Oncol. (2021) 23:iii1iii105. doi: 10.1093/neuonc/noab200 34608945 PMC8491279

[2] Bunin GR Surawicz TS Witman PA Preston-Martin S Davis F Bruner JM. The descriptive epidemiology of craniopharyngioma. J Neurosurg. (1998) 89:547–51. doi: 10.3171/jns.1998.89.4.0547 9761047

[3] Bollati A Giunta F Lenzi A Marini G. Third ventricle intrinsic craniopharingioma. Case Rep J Neurosurg Sci. (1974) 18:216–9. 4465421

[4] Prieto R Barrios L Pascual JM. Papillary craniopharyngioma: A type of tumor primarily impairing the hypothalamus - A comprehensive anatomo-clinical characterization of 350 well-described cases. Neuroendocrinology. (2022) 112:941–65. doi: 10.1159/000521652 35108706

[5] Karavitaki N Brufani C Warner JT Adams CB Richards P Ansorge O. Craniopharyngiomas in children and adults: systematic analysis of 121 cases with long-term follow-up. Clin Endocrinol (Oxf). (2005) 62:397–409. doi: 10.1111/j.13652265.2005.02231.x 15807869

[6] Garrè ML Cama A. Craniopharyngioma: modern concepts in pathogenesis and treatment. Curr Opin Pediatr. (2007) 19:471–9. doi: 10.1097/MOP.0b013e3282495a22 17630614

[7] Juratli TA Jones PS Wang N Subramanian M Aylwin SJB Odia Y. Targeted treatment of papillary craniopharyngiomas harboring BRAF V600E mutations. Cancer. (2019) 125:2910–4. doi: 10.1002/cncr.32197 PMC7032527 31314136

[8] Yamada S Fukuhara N Yamaguchi-Okada M Nishioka H Takeshita A Takeuchi Y. Therapeutic outcomes of transsphenoidal surgery in pediatric patients with craniopharyngiomas: a single-center study. J Neurosurg Pediatr. (2018) 21:549–62. doi: 10.3171/2017.10.PEDS17254 29600905

[9] Brastianos PK Twohy E Geyer S Gerstner ER Kaufmann TJ Tabrizi S. BRAF-MEK inhibition in newly diagnosed papillary craniopharyngiomas. N Engl J Med. (2023) 389:118–26. doi: 10.1056/NEJMoa2213329 PMC10464854 37437144

[10] Müller HL Merchant TE Warmuth-Metz M Martinez-Barbera JP Puget S. Craniopharyngioma. Nat Rev Dis Primers. (2019) 5:75. doi: 10.1038/s41572-019-0125-9 31699993

[11] Poretti A Grotzer MA Ribi K Schönle E Boltshauser E. Outcome of craniopharyngioma in children: long-term complications and quality of life. Dev Med Child Neurol. (2004) 46:2209. doi: 10.1017/s0012162204000374 15077699

[12] Omholt K Platz A Kanter L Ringborg U Hansson J. NRAS and BRAF mutations arise early during melanoma pathogenesis and are preserved throughout tumor progression. Clin Cancer Res. (2003) 9:6483–8. doi: 10.1158/1078-0432.ccr-03-0884 14695152

[13] Salama AKS Li S Macrae ER Park JI Mitchell EP Zwiebel JA. Dabrafenib and trametinib in patients with tumors with BRAFV600E mutations: results of the NCI-MATCH trial subprotocol H. J Clin Oncol. (2020) 38:3895–904. doi: 10.1200/JCO.20.00762 PMC7676884 32758030

[14] Gershenson DM Miller A Brady WE Paul J Carty K Rodgers W. Trametinib versus standard of care in patients with recurrent low-grade serous ovarian cancer (GOG 281/LOGS): an international, randomised, open-label, multicentre, phase 2/3 trial. Lancet. (2022) 399:541–53. doi: 10.1016/S0140-6736(21)02175-9 PMC8819271 35123694

[15] Brastianos PK Shankar GM Gill CM Taylor-Weiner A Nayyar N Panka DJ. Dramatic response of BRAF V600E mutant papillary craniopharyngioma to targeted therapy. J Natl Cancer Inst. (2015) 108:djv310. doi: 10.1093/jnci/djv310 26498373 PMC4862417

[16] Roque A Odia Y. BRAF-V600E mutant papillary craniopharyngioma dramatically responds to combination BRAF and MEK inhibitors. CNS Oncol. (2017) 6:95–9. doi: 10.2217/cns-20160034 PMC6020871 28425764

[17] Nussbaum PE Nussbaum LA Torok CM Patel PD Yesavage TA Nussbaum ES. Case report and literature review of BRAF-V600 inhibitors for treatment of papillary craniopharyngiomas: A potential treatment paradigm shift. J Clin Pharm Ther. (2022) 47:826–31. doi: 10.1111/jcpt.13600 35023192

[18] Wu ZP Wang YL Wang LC Liu ZY Fan RR Zan X. Case report: successful use of BRAF/MEK inhibitors in aggressive BRAF-mutant craniopharyngioma. World Neurosurg. (2023) 180: e117–26. doi: 10.1016/j.wneu.2023.08.137. ahead of print. 37683921

[19] Rao M Bhattacharjee M Shepard S Hsu S. Newly diagnosed papillary craniopharyngioma with BRAF V600E mutation treated with single-agent selective BRAF inhibitor dabrafenib: a case report. Oncotarget. (2019) 10:6038–42. doi: 10.18632/oncotarget.27203 PMC6800270 31666933

[20] Rostami E Witt Nyström P Libard S Wikström J Casar-Borota O Gudjonsson O. Recurrent papillary craniopharyngioma with BRAFV600E mutation treated with neoadjuvant-targeted therapy. Acta Neurochir (Wien). (2017) 159:2217–21. doi: 10.1007/s00701-017-3311-0 PMC5636852 28918496

[21] Khaddour K Chicoine MR Huang J Dahiya S Ansstas G. Successful use of BRAF/MEK inhibitors as a neoadjuvant approach in the definitive treatment of papillary craniopharyngioma. J Natl Compr Canc Netw. (2020) 18:1590–5. doi: 10.6004/jnccn.2020.7624 33285519

[22] Garutti M Bergnach M Polesel J Palmero L Pizzichetta MA Puglisi F. BRAF and MEK inhibitors and their toxicities: A meta-analysis. Cancers (Basel). (2022) 15:141. doi: 10.3390/cancers15010141 36612138 PMC9818023

