# Supplementary figures and images for: Clinical response to dabrafenib plus trametinib in BRAF V600E mutated papillary craniopharyngiomas: a case report and literature review
Source: Front Oncol. 2024 Nov 27;14:1464362. doi: 10.3389/fonc.2024.1464362 (PMC11631891; doi:10.3389/fonc.2024.1464362)

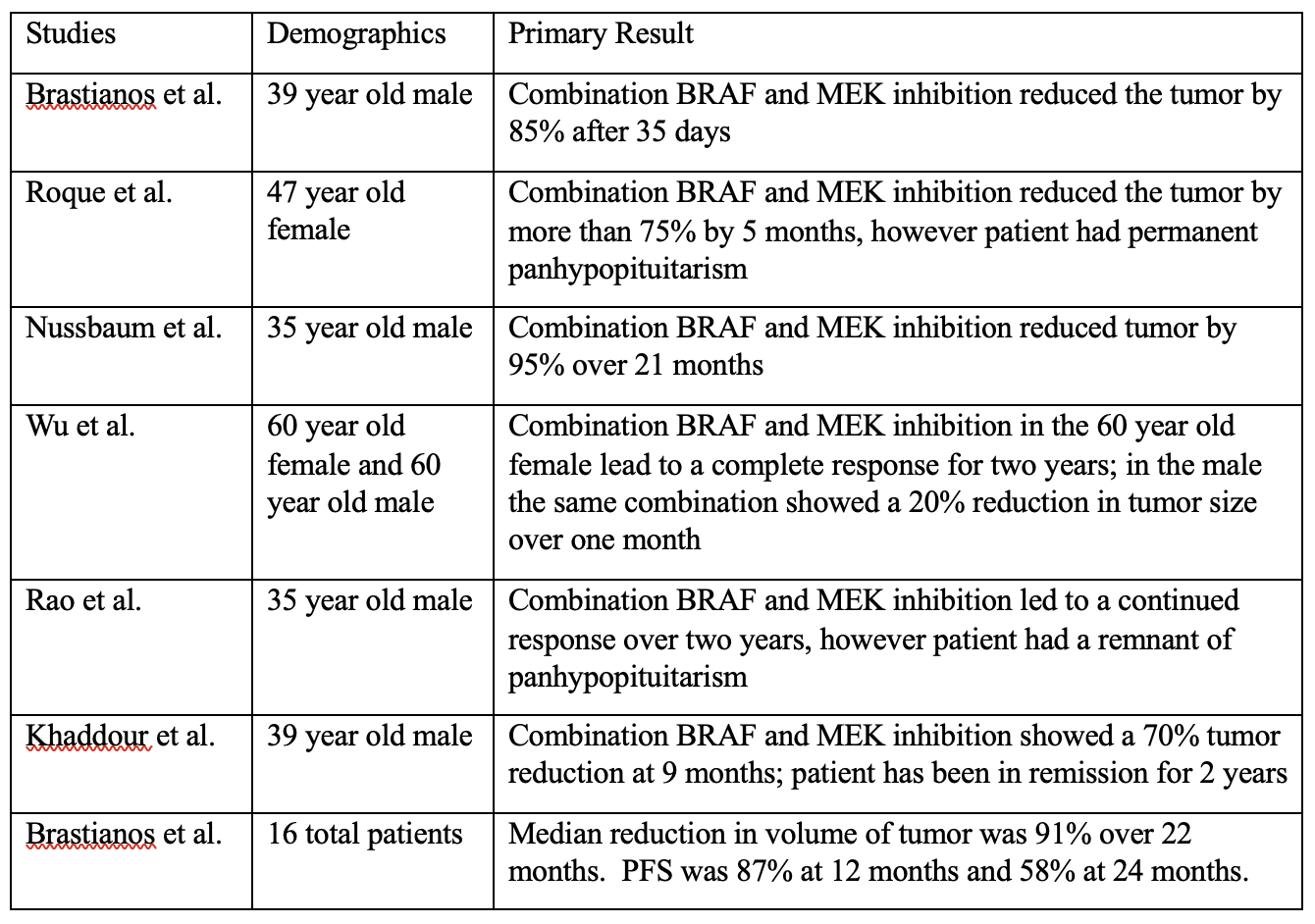

Supplement: Supplementary file 1 [file Image1.tif]
